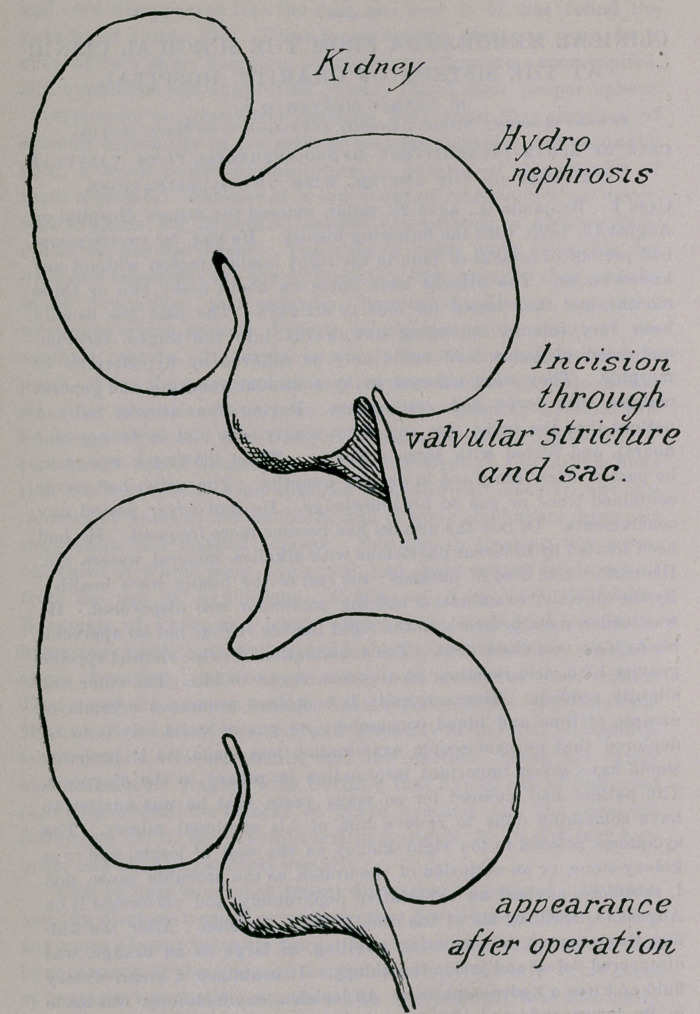# Clinical Memoranda from the Surgical Clinic at the Sisters’ of Charity Hospital

**Published:** 1893-11

**Authors:** Herman Mynter

**Affiliations:** Professor of Surgery, Niagara University, and Surgeon to the Sisters’ Hospital


### CLINICAL MEMORANDA FROM THE SURGICAL CLINIC AT THE SISTERS' OF CHARITY HOSPITAL.

By HERMAN MYNTER, M. D.,
Professor of Surgery, Niagara University, and Surgeon to the Sisters' Hospital.

### CASE OF ACUTE INTERMITTENT HYDRO-NEPHROSIS, FROM VALVULAR STRICTURE OF THE URETER, WITH TWO ILLUSTRATIONS.

Case I.—Benjamin G., aged 25, tailor, entered the Sisters' Hospital on August 12, 1893, with the following history : He had, for twelve years, had periodical attacks of pain in the right lumbar region without any known cause. The attacks have come on about every two or three months, and then lasted for four or six days. The pain has usually been very intense, extending downwards into the thighs, scrotum, and head of penis, and could only be relieved by hypodermics of morphia. They were ushered in by continual vomiting and general malaise, with fever and restlessness. During the attacks he had noticed that the urine was somewhat scanty (one pint in twenty-four hours), and voided with some difficulty. When the attack was over, he passed urine freely and in greater quantity. The urine had never contained blood or pus to his knowledge. He had never passed any concrements. Of late the attacks had become more frequent. He had been treated by different physicians with alkalies, mineral waters, etc. His mother had died of phthisis ; the rest of the family were healthy. By the objective examination nothing particular was discovered. He was tender, upon pressure, in the right lumbar region, but no appreciable fullness was discovered. The examination of urine showed specific gravity 1035, acid reaction, no albumen, sugar, or bile. The color was slightly reddish. Microscopically it contained numerous crystals of oxalate of lime and blood corpuscles ; no pus or casts. It is to be deplored that no cystoscopic examination was made, as it probably would have given important information in regard to the diagnosis. The patient had suffered for so many years, that he was anxious to have something done to relieve him of his continual misery. The symptoms pointed to the right kidney as the seat of lesion, and to a kidney-stone, or an occlusion of the ureter, as the probable cause, and I, therefore, advised an explorative nephrotomy, and performed it on August 14, 1893, by aid of the usual oblique incision. After the kidney was exposed, a fluctuating swelling, as large as an orange, was discovered below and inside the kidney. It contained a clear, watery fluid and was a hydro-nephrosis. An incision, one inch long, was made in its lower end, and about one-half pint of fluid evacuated. The finger was introduced and the pelvis explored for stone, but none were

Kidney

Hydro nephrosis

Incision through valvular stricture and sac.

appearance after operation found. By spreading the incision laterally the opening of the ureter could be seen plainly. It appeared as a papilla, extending one-fourth of an inch into the cavity. A flexible bougie, No. 14 French scale, was introduced with ease into the bladder, showing the ureter to be permeable through its whole length. I could find no other cause for the recurrent hydro-nephrosis than this abnormal condition of the ureter. The kidney was not more movable than normally. I, therefore, enlarged the incision downwards through the papilla and well into the healthy ureter, pulled the margins of the wound outwards with fine hooks, and united the wound with numerous fine silk sutures, taking in the outer two coats of the ureter and the sac and avoiding the mucous membrane. After the wound was sutured, the appearance was more like that of a funnel. (See illustrations.) The wound in the ureter and sac was protected with a mesh of iodoform gauze for possible drainage, and the rest of the wound closed. For three or four daysa fter the operation he complained of considerable tenderness in the lumbar region, and moderate fever. No discharge of urine occurred through the wound. The urine contained considerable blood, and had to be drawn by catheter. The amount was seventeen and eighteen ounces the first and second days, twenty ounces the third day, twenty-six ounces the fourth, thirty-eight ounces the sixth, and thereafter about forty ounces daily. Under the use of

Tinct. chlor. iron.................................gtt. 20

Fld. ext. ergot....................................ʒss.

Acid gallici.........................................gr. x.

Glycerine, q. s. ad.................................℥ss.

d. Every four hours.

the urine cleared up and became normal, all pain and tenderness disappeared, the wound healed, and on August 29th, fifteen days after the operation, he was discharged well. He has since been well, and had no attacks. From a letter of October 11th, I quote: "I am still feeling well, and hardly realize that there ever was an operation performed on me." I have so far not made a cystoscopic examination, and satisfied myself that the function of the kidney is restored.

This case is of interest in more than one way. It proves, what otherwise is well known, that incised wounds of the ureter may heal as any other wound if carefully sutured. Fenger, of Chicago, has published a very similar case (*Chicago Medical Recorder*, March, 1893.) He remedied the defect by dividing the valve transversely and uniting the ends of the incision by suture. In a case of stricture lower down he made a longitudinal incision and united it transversely, similar to the operation of Heinecke-Mikulicz for stenosis of the pyloris. I united the incision by longitudinal suture in my case, as there seemed to be a redundance of tissue after the tip of the valve had been pulled outwards.

Fenger states that "valvular stricture at the pelvic orifice of the ureter is usually caused by lateral insertion of the ureter in a dilated pelvis." Küster, in a similar case, but with another stricture lower down, resected the stricture and united the ureter with the pelvis of the kidney. The result was excellent, but, as Fenger states, the plastic operation is easier of technique.

The spontaneous evacuation of the hydro-nephrosis was probably due to obliteration of the valve, or papilla, by pressure, when the hydro-nephrosis reached a certain degree.

The usual cause of intermittent hydro-nephrosis (according to Terrier and Baudonin, who publish eighty-three cases,) is a floating kidney, causing a kink in the ureter, and thus arresting the evacuation of urine.

Most of the cases become, eventually, permanent by inflammatory changes, which form bands of adhesions and thus fasten the kidney in its displaced position. They advise early nephroraphy, or else nephrectomy. Judging from the successful result of Fenger's and my own cases, nephrectomy can scarcely be indicated. It is well enough to call the attention of surgeons to the possibility that a valvular stricture may be the cause of the acute hydro-nephrosis, whether there be a floating kidney or not, and that it then may be remedied. by nothing more serious than a slight plastic operation.

In the January (1893) number of the BUFFALO MEDICAL AND SURGICAL JOURNAL I reported eleven cases of operations on the kidney for various lesions. Three of these cases were nephroraphies for floating kidneys. Although considerably benefited by the operation, the result was not what had been desired. Sutures of animal tissue had been used ; and what is there to prevent prolapse of the kidney again after the sutures have been absorbed ? If too tightly knotted they will cut out anyway. Riedel tried to improve the results by producing adhesions between the kidney and the diaphragm, and he was successful in five cases. Having exposed the kidney, he strips off the fibrous capsule and pushes the organ so far up behind the diaphragm that only its lower half is exposed. The median portion of the fibrous capsule is then fixed to the anterior portion of the quadratus lumborum muscle by deep catgut sutures. A strip of iodoform gauze is then thrust up between the kidney and the diaphragm, so that the entire posterior surface of the kidney is covered, a second piece of iodoform gauze is introduced into the space formerly occupied by the prolapsed kidney, and a third piece is placed upon the lower portion of the kidney lying upon the anterior surface of the quadratus lumborum muscle. The whole wound is thereafter sutured, leaving the ends of the gauze projecting at its upper and lower ends, and the gauze tampons kept in place for four weeks. When then removed, the cavity between the kidney and the diaphragm will be found surrounded with strong, healthy granulations, which will be changed into fibrous tissue and firm, strong adhesions between the kidney and diaphragm.

I carried this operation out in the following two cases :

CASE II.—Nephroraphy for floating kidney, after Riedel's method. Mrs. H., 32 years old, had been in perfect health till April, 1891, when she commenced to complain of dragging pain in the right side of the abdomen. She emaciated rapidly, her weight falling from 118 pounds to ninety-five pounds, complained of loss of appetite, insomnia, headache, and frequent attacks of renal colic. A tender and movable lump was discovered in the lower part of the abdomen. Shortly after she became pregnant, and as the pregnancy advanced, the symptoms gradually disappeared, so that she "had never felt better in her life." After confinement the symptoms returned with increased severity, the nervous symptoms particularly being severe. Dr. Frederick, who had delivered her, then discovered a floating kidney near the brim of the pelvis, which could be moved about with great ease, but was very tender on pressure. On January 17, 1893, I performed nephroraphy, after Riedel's method. With the exception of some surgical fever during the first days, and considerable oozing of serum from the wound, necessitating large dressings, the course was uneventful. On February 18th, the gauze tampons were removed, and the kidney and surrounding tissues found studded with healthy granulations. Drainage tubes were introduced. March 8th, the tubes were removed. March 12th, wound healed. March 17th, patient allowed to get up ; kidney feels immovably fixed. September 23, 1893, the patient has since felt well, eats and sleeps well, has no pain, and is able to do all her housework and take care of her two children. The kidney cannot be felt in any position.

CASE III.—Nephroraphy for floating kidney, after Riedel's method. Mrs. R., 45 years of age, had for six months complained of severe pain one and a half inches to the right of the ensiform cartilage. The pain would come periodically, and be attended with yellow color of skin and conjunctivæ and clay-formed stools. Five weeks previous to her entrance in the hospital she had symptoms indicating peritonitis, followed by a very severe attack of pain and jaundice, lasting four days. She felt then a lump in right hypochondriac region of the size of an egg, and corresponding to the gall-bladder. She entered in order to have a cholecystotomy performed, it being supposed, from the history and symptoms, that she suffered from gall-stone colic. Over the region of the gall-bladder a pear-formed, nodulated, apparently immovable tumor was felt, which had all the appearances of an indurated gall-bladder filled with gall-stones. An explorative incision was made in September, 1892, along the right margin of the rectus muscle, but on entering the abdominal cavity, much to our surprise, no tumor was found, and even the gall-bladder was absent. A hard lump was felt behind the colon transversum. An opening having been torn through the mesentery of the colon transversum, it was found to be a floating kidney. The wound was therefore closed, and the patient left the hospital in two weeks. She felt well for three months, when the attacks returned more severe than ever, and could only be controlled by opiates in large doses. For the last two weeks she had noticed that previous to, and during the attack, there was a decreased amount of urine, and that just after the attack had passed away, the urine would be discharged in large quantity, all indicating acute hydronephrosis from a kink of the ureter. She entered the hospital again on June 26, 1893. The kidney was then felt freely movable over the brim of the pelvis, moving over an area of about three or four inches. June 26th, operation after Riedel's method. The further course was favorable. The tampons were removed on July 25th, and the patient left the hospital on August 14th. October 3, 1893, the patient has had no attacks since, and says she is feeling perfectly well. She has gained considerably in flesh. There is no tenderness, even by deep pressure, in the iliac region, and the kidney cannot be felt in any position.

---

THE Massachusetts Institute of Technology has published a beautiful and handsomely illustrated brochure, giving a brief account of its foundation, character, and equipment, that was prepared in connection with the World's Columbian Exposition. It is published by the institute, and printed by the University Press, Cambridge. It contains much interesting information relating to this remarkable educational institution, and well repays reading by every friend of advanced education in this country. It can be obtained on application to Dr. H. W. Tyler, Secretary Institute of Technology, Boston, Mass.

## Figures and Tables

**Figure f1:**